# IL-17A inhibitors alleviate Psoriasis with concomitant restoration of intestinal/skin microbiota homeostasis and altered microbiota function

**DOI:** 10.3389/fimmu.2024.1344963

**Published:** 2024-02-28

**Authors:** Huixia Zhao, Lili Shang, Yuting Zhang, Zhaojun Liang, Nan Wang, Qian Zhang, Chong Gao, Jing Luo

**Affiliations:** ^1^ Department of Dermatology, Heji Hospital of Changzhi Medical College, Changzhi, China; ^2^ Department of Rheumatology, The Second Clinical Medical College of Shanxi Medical University, Taiyuan, China; ^3^ Shanxi Key Laboratory for immunomicroecology, The Second Hospital of Shanxi Medical University, Taiyuan, China; ^4^ Department of Rheumatology, The Second Hospital of Shanxi Medical University, Taiyuan, China; ^5^ Brigham and Women’s Hospital, Harvard Medical School, Boston, MA, United States

**Keywords:** psoriasis, gut microbiome, skin microbiome, gut-skin axis, IL-17A inhibitor

## Abstract

**Background:**

Disturbed gut microbiota and associated metabolic dysfunction exist in Psoriasis. Despite the growing use of interleukin-17 inhibitor (anti-IL17) therapy, the effect of anti-IL17 on gut/skin microbiota function is not fully understood in patients with Psoriasis.

**Objective:**

Therefore, we explored whether Psoriasis is associated with alterations in selected gut/skin microbiota in a study cohort, and a longitudinal cohort study to reveal the effects of IL-17A inhibitor treatment on gut microbiota in Psoriasis.

**Methods:**

In a case-control study, 14 patients with Psoriasis and 10 age, sex and body mass index-matched Healthy Controls were recruited. Longitudinal mapping of the gut microbiome was performed using 16S rRNA gene sequencing. Mouse models were used to further study and validate the interrelationship between the skin microbiome and the gut microbiome in Psoriasis. PICRUST2 was applied to predict the function of the bacterial community.

**Results:**

In Psoriasis patients, gut microbiota dysbiosis was present with increased heterogeneity: decreased *Bacteroidota* and increased *Firmicutes* as well as *Actinobacteriota* predominating in Psoriasis. *Escherichia-Shigella* enrichment was associated with reduction in serum levels of total bile acid and markers in Apoptotic pathways. After IL-17A inhibitor treatment in Psoriasis patients, longitudinal studies observed a trend toward a normal distribution of the gut microbiome and modulation of apoptosis-related metabolic pathways. Results from a mouse model showed dysregulation of the skin microbiota in Psoriasis characterized by *Staphylococcus* colonization.

**Conclusion:**

The psoriatic gut/skin microbiota exhibits loss of community stability and pathogen enrichment. IL-17A inhibitors restore microbiota homeostasis and metabolic pathways, reduce pro-inflammatory cytokine expression, and alleviate symptoms in patients with Psoriasis.

## 1 Introduction

Psoriasis (PSO) is a chronic immune-mediated inflammatory disease that affects approximately 2-4% of the population worldwide (1). While it is primarily characterized by skin involvement, it can also cause systemic inflammation in various organs (2). PSO can be highly debilitating (3) and can increase the morbidity and mortality associated with the disease (4). The development of PSO involves a combination of genetic, immunological, and environmental factors (5). An overactive Th17 response can mediate skin inflammation and hyperproliferation of keratinocytes in PSO (6). Although therapies targeting the Th17 pathway can effectively suppress the abnormal immune response and manage symptoms in PSO patients, they do not provide a cure, and the effectiveness of treatment can vary from patient to patient. This emphasizes the need to further understand the factors involved in the development and progression of PSO.

The interaction between the gut microbiome and inflammatory skin diseases is mediated by a dysfunctional gut microbiome, a disrupted gut barrier, increased inflammatory mediators, and metabolites produced by microorganisms (7–9). Gut microecological dysregulation activates a pro-inflammatory state by altering the metabolic environment and activating specific pattern recognition receptors (PRPs) on epithelial cells, which alters the integrity of the tight junctions between epithelial cells, leading to increased permeability of the intestinal, increased release of proinflammatory cytokines, exacerbation of chronic inflammation, and the entry of certain metabolites, toxins, and even bacteria into the systemic circulation (10, 11), and when these microorganisms and their metabolites enter the circulation they can either promote or maintain the proinflammatory state in an organism (12). Polysaccharide A produced by *B. fragilis* binds to TLR-2 and promotes the differentiation of CD4^+^ lymphocytes to Treg lymphocytes, along with the production of anti-inflammatory cytokines such as IL-10 (13). *Segmented Filamentous Bacteria*, as a commensal bacterium, influence the adaptive and innate immune systems by increasing IgA secretion and the development of Th17 lymphocytes (14). Butyrate, produced by intestinal bacteria, promotes the differentiation of Treg cells and has anti-inflammatory properties (15). Elevated claudin 3 has been observed in PSO patients, suggesting increased intestinal permeability (12). Because overexpression of proinflammatory cytokines leading to an imbalance between effector T cells and epithelial permeability, gut microecological dysregulation contributes to PSO-associated inflammatory and autoimmune states (10).

The skin, being the body’s largest organ, serves as a physical barrier against environmental damage and as a dynamical interface for host-skin-microbe interactions. Skin bacteria play a vital role in maintaining skin health by preventing pathogen colonization and regulating T lymphocyte function (16). Keratinocytes can interact with skin bacteria to trigger an immune response in PSO (17). Therefore, disruptions to the skin’s microbiome can potentially affect skin’s immune function. The gut microbiome also influences skin homeostasis by impacting the signaling processes involved in epidermal differentiation (18). The number of *Firmicutes*, *Proteobacteria*, and *Actinobacteria* in PSO has increased. Decreased abundance of *Bacteroides* in PSO patients indicating that decreased abundance of beneficial microorganisms may disturb skin homeostasis by altering the balance of the immune system (19). Inflammatory mediators, such as IFN-γ, IL-1β, IL-6, IL-12, and TNF, are significantly increased in patients with PSO (20, 21). Additionally, genetic material from intestinal bacteria has been discovered in plasma samples from PSO patients, indicating a potential connection between intestinal microorganisms and psoriatic skin lesions (22).

Current immunologic studies have revealed the important role of immune pathways involving interleukin (IL)-17 in PSO (23). *Bacillus hominis (Bu)* has been found to attenuate colitis by modulating host Bile Acids (BAs) metabolism, impacting the IL-17 signaling pathway, and inhibiting Th17 cell differentiation (24). *Lactobacillus pentosus* decrease the suppression of pro-inflammatory cytokines, such as TNF-α, IL-17A, and IL-23 (25). The interaction between the microbiota and the immune system holds clinical significance. The efficacy of Secukinumab, an IL-17 inhibitor, has been demonstrated in the treatment of PSO (26), and studies have suggested that anti-PSO therapy may influence the composition of the microbiota (27).

In this study, we examined and observed the alterations in gut microbiomes in 14 PSO patients compared to 10 healthy subjects using 16S rRNA sequencing. Through a longitudinal observation of Secukinumab treatment, we observed a trend of abnormal changes in the gut microbiomes of PSO patients returning to a more normal state. Additionally, we utilized animal models to identify *Staphylococcus* as a key factor in PSO skin lesions.

## 2 Material and methods

### 2.1 Study design and patients

**Figure 1:**
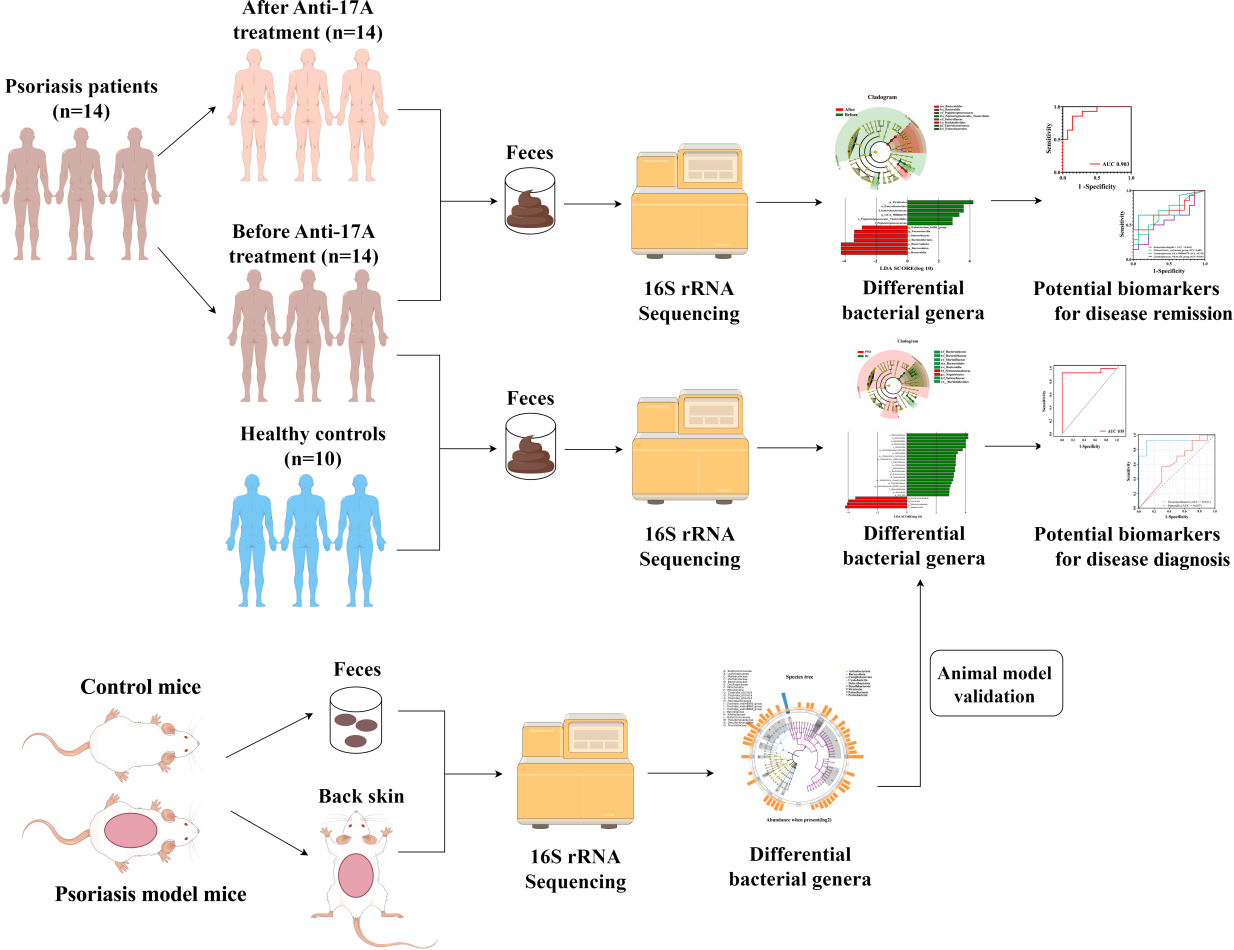
Experimental flow chart.

A total of 14 patients diagnosed with Psoriasis (PSO) were included in this study (**Figure 1**). They were treated with Secukinumab, an interleukin-17 inhibitor, at the Department of Dermatology, Heji Hospital of Changzhi Medical College, from September 2022 to May 2023. All patients had a clear diagnosis of PSO and followed the Guideline for the diagnosis and treatment of psoriasis in China (2018 complete edition) (28). Among them, 2 patients were newly diagnosed, while the other 12 had a previous diagnosis but had not received treatment with hormonal drugs, immunosuppressants, or biologics within the past year. Ten healthy controls (HCs) were also included, matched in terms of age and sex, and had no history of psoriasis, autoimmune diseases, tumors, and gastrointestinal tract disorders, or any other disease that could affect the results of the study were excluded. All included subjects have inclusion criteria: (1) normal range of liver and kidney functional tests; (2) normal blood glucose/lipid, normal urine and stool; (3) free of hepatitis B/C virus antigen; and (4) had no history of antibiotics and probiotics during the past 4 weeks (**Supplementary Table 1**).

The PSO patients in the anti-IL-17 drug group received secukinumab 300 mg at weeks 0, 1, 2, 3, and 4, followed by administration every 4 weeks. Baseline demographic and clinical data, including age, gender, and laboratory markers, were collected. Disease severity was assessed using the Psoriasis Area and Severity Index (PASI) scores (29), with assessments conducted at Weeks 0 and 16 by the same physician. A PASI 75 score indicates a 75% or greater reduction in PASI score from baseline, representing significant disease improvement. This criterion has been traditionally used as a benchmark for treatment efficacy in clinical trials (30, 31). Therefore, respondents are defined as patients who have improved PASI by ≥ 75% after 16 weeks of treatment, whereas non-responders were defined as <75% improvement in PASI. The study received ethical approval from the Ethics Committee of Heji Hospital of Changzhi Medical College (number: 2023-13), and all patients provided written informed consent to participate.

### 2.2 Construction of Psoriasis mouse model

Male BALB/c mice (SPF grade, aged 5 weeks, weighing 16 ~ 18 g) were obtained from Beijing Spectrum Biotechnology Co. and housed at a temperature of 23°C and a humidity level of 50%. All animal procedures were approved by the Ethics Committee of Changzhi Medical College (number: DW2023051).

After one week of acclimatization, the mice were randomly assigned to two groups: experimental and control, with six mice in each group. Hair on the dorsal area of all mice was removed using depilatory cream. Mice in the experimental group were topically treated with imiquimod cream (IMQ, Sichuan Mingxin Pharmaceutical Co., Ltd., 3 g/tube, [Approval Number] National Drug License H20030129) at a dose of 62.5 mg/day on their dorsal skin for seven consecutive days, while control mice received an equivalent volume of petroleum jelly (Shandong Dezhou Likang Pharmaceutical Science and Technology Co., Ltd., 300 g/box) for the same duration. Throughout the experiment, the experimental and control groups were maintained under identical conditions, with no significant differences in diet, water access, ambient temperature, or humidity. On the 8th day, the mice were euthanized following anesthesia.

### 2.3 Sample collection

#### 2.3.1 Stool sample collection

Stool specimens were obtained from subjects at baseline (M0) and after 16 weeks of PSO treatment with the anti-IL-17A medication (Secukinumab). The collected specimens were then placed in pre-labeled stool collection tubes containing fecal DNA stabilizer. After blending through shaking, the specimens were immediately stored at -80°C in frozen storage until transportation on ice to the laboratory for further processing.

On the 8th day of mouse modeling, fresh fecal samples were collected from both groups of mice. Two grams of fecal samples were dispensed into sterile freezing tubes immediately after sampling and placed in a -80°C refrigerator for freezing and storage. The collection process and requirements remained unchanged.

#### 2.3.2 Skin tissue sample collection

On the 8th day of conducting mouse modeling, the mice were subjected to anesthesia using a carbon dioxide gas anesthesia machine (PerkinElmer). Skin tissue samples measuring approximately 1cm×1cm×2cm were obtained from the back of the mice and labeled as No. 1 and No. 2 samples. Following the sampling procedure, sample No. 1 was fixed in a sterile formalin solution, while sample No. 2 was placed in a sterile freezing tube and stored in a -80°C refrigerator for freezing. All samples were collected by the same laboratory personnel, and strict adherence to sterility principles was maintained to prevent contamination of the experimental specimens by other microorganisms during the collection process.

### 2.4 DNA extraction and 16S rRNA gene sequencing

Fecal samples were collected at the baseline and 16 weeks after treatment using the Longsee Fecalpro kit (Longsee Medical Technology Co., Guangzhou, China). DNA extraction was performed using a nucleic acid extraction or purification reagent (Hangzhou Guhe: GHFDE100) according to the manufacturer’s instructions. The quantity and quality of the extracted DNA were assessed using a NanoDrop ND-1000 spectrophotometer (Thermo Fisher Scientific, Waltham, MA, USA). The variable regions 3 and 4 (V3-V4) of the 16S rRNA gene, PCR mixture conditions, and the corresponding thermal cycling steps were amplified by PCR using primer sets (Illumina V3 forward 5′-CCTACGGGGNGGCWGCAG-3′ and V4 reverse 5′-GACTACHVGGGTATCTAATCC′). The purified libraries were quantified, normalized, and sequenced using a NovaSeq instrument (Illumina).

### 2.5 Sequencing data processing and species annotation

The reads from each sample were spliced using Vsearch v2.15.0 in order to obtain the raw Tags data (Raw Tags). Sequence quality was also controlled and filtered. Information was collected from the SILVA database and a pre-fitted QIME2 classifier built with the Scikit Lean package was applied to assign bacterial classifications. Filtered OTU sequences were used in PICRUSt prediction, with default parameters.

### 2.6 PICRUSt2 functional prediction analysis

Phylogenetic studies of the cohort were performed by reconstructing the unobserved state 2 (PICRUST2) to predict the functional potential of the bacterial community. R (v3.6.1) was utilized for the generation of other graphs.

### 2.7 Statistical analysis

Alpha diversity and beta diversity were calculated using QIME2. Principal Coordinate Analysis (PCoA) was conducted based on the UniFrac distance metric. Subsequently, Wilcoxon tests, linear discriminant analysis (LDA) effect size (LEfSe) analyses, and metagenomeSeq analyses were performed to identify the differential classification group. All statistical tests were two-tailed, and p-values less than 0.05 were considered statistically significant.

## 3 Results

### 3.1 Patient cohort

The study included a cohort of 14 patients diagnosed with PSO and 10 HCs, among which 5 had guttate psoriasis and 9 had plaque psoriasis (**Supplementary Table 2**). Two groups of subjects, matched for sex and age, were compared (**Table 1**; **Supplementary Table 3**). All PSO patients were diagnosed at the Dermatology Department of Changzhi Heji Hospital. The average PASI score for the PSO patients in this study was 22.04, indicating that all enrolled subjects had moderate to severe disease. To minimize treatment-related variability, subjects who had recently undergone antibiotic therapy and/or other biological and systemic therapies were excluded.

**Table 1:** Demographic and clinical characteristics of the study participants.

	Psoriasis (n=14)	Healthy Controls (n=10)	*P* value
Demographic			
Age (years)	38.64 ± 10.46	49.10 ± 11.13	0.091
Female/male, n	4/10	7/3	
Disease duration (years)	14.36 ± 6.44	–	
Laboratory parameters			
WBC (*10^9^/L)	7.23 ± 2.46	6.57 ± 1.39	0.448
Hemoglobin (g/L)	148.86 ± 16.52	147.7 ± 16.57	0.867
PLT (*10^9^/L)	246.8 ± 119.49	291.4 ± 43.83	0.274
Neutrophile (*10^9^/L)	4.98 ± 2.48	4.15 ± 1.42	0.352
Lymphocyte (*10^9^/L)	1.76 ± 0.51	2.08 ± 0.53	0.157

Mean (x ± s). Intergroup and Within-group comparisons of duration were made using the Independent-Samples T Test. Intergroup and Within-group comparisons of age were made using Pearson Chi-square test. WBC, White blood cell; PLT, Platelet.

### 3.2 Altered gut microbial diversity in Psoriasis patients

**Figure 2:**
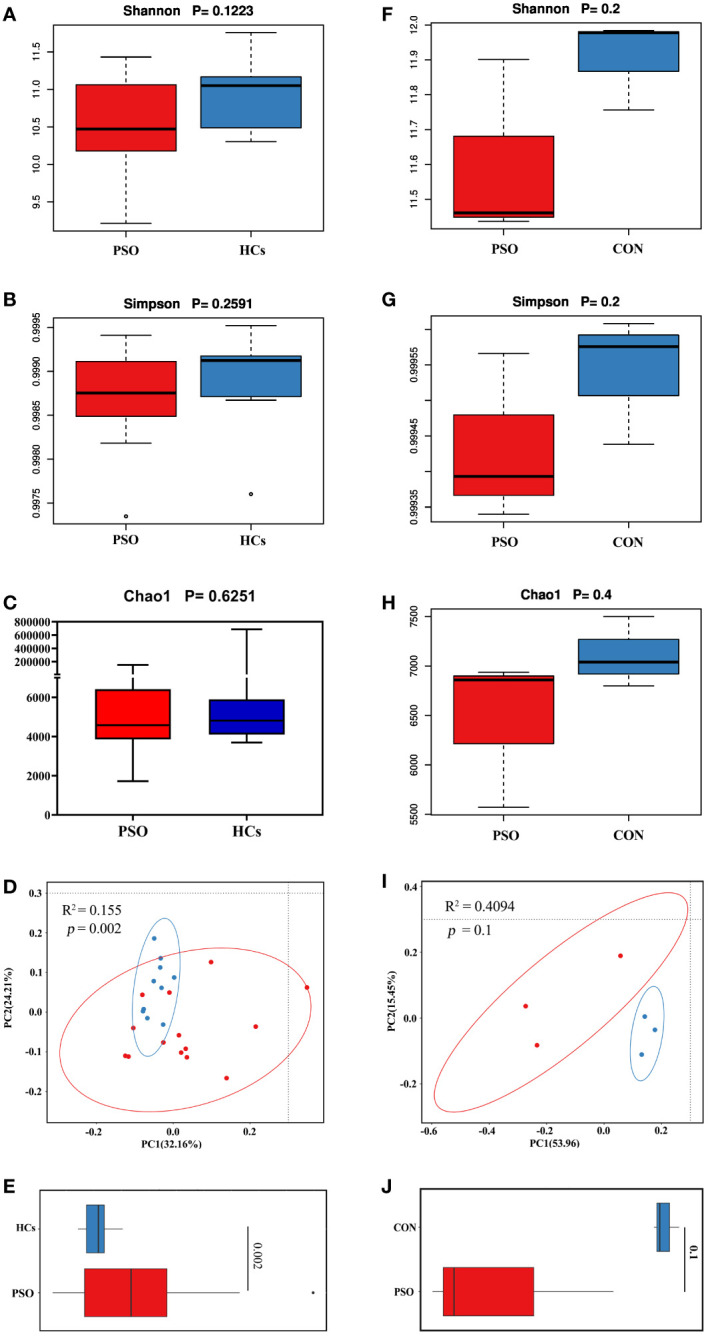
Bacterial community diversity in the healthy and psoriatic gut. Alpha diversity measured according to **(A)** Shannon index, **(B)** Simpson’s diversity index, and **(C)** chao1 index of healthy skin samples. **(D)** Principal coordinate analysis (PCoA) of the microbial community structures based on weighted UniFrac distance matrix for the first two principal axes. Each point on the PCoA plot represents a fecal microbiome sample (red = HCs, blue = PSO). The first principal coordinate explains 32.16% of variation, and the second principal coordinate explains 24.21% of the variation. **(E)** The average weighted UniFrac distances among samples within each state are shown in the box plot. **(F-H)** Alfa diversity of the gut microbiome in PSO model mouse. **(I, J)** Beta diversity of the gut microbiome in PSO model mouse (the average unweighted UniFrac distances). HCs: Healthy Controls. PSO: Psoriasis patients and psoriasis model mouse, CON, Control Mouse.

Differences in the overall composition of the microbial community between PSO patients and controls were assessed by measuring α- and β-diversity. Alpha diversity reflects the microbial diversity within a sample, while beta diversity captures differences between samples. It was observed that, although not statistically significant, the gut microbiome in PSO patients exhibited reduced α-diversity (**Figures 2A-C**). This trend suggests that the diversity of the gut microbial community becomes homogenous as PSO progresses.

Beta diversity was calculated using the weighted UniFrac metric to determine whether the structure of the microbial community differed between psoriatic subjects and HCs. Principal coordinates analysis revealed significant differences in the gut microbiome between PSO patients and HCs, with the former showing greater dispersion (**Figure 2D**). PSO patients also exhibited higher mean distances in the composition of their gut microbiota compared to HCs (**Figure 2E**), indicating greater heterogeneity. Consistent results were also observed in a mouse model (**Figures 2F-J**).

### 3.3 Changes in gut microbial composition in Psoriasis

**Figure 3:**
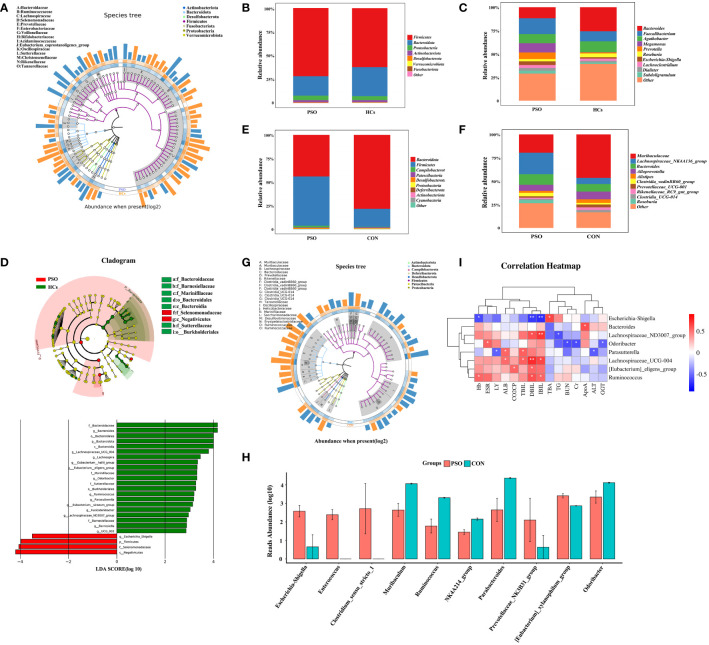
Taxonomic composition and gut microbial characteristics associated with disease states. **(A)** GraPhlan diagram showing the dominant flora in PSO. **(B)** Phylum and **(C)** genus level compositions of gut microbiome in HCs and PSO. Only major taxa are shown. Others represent less abundant taxa and are not plotted. **(D)** Results of LEfSe analysis of variance. **(E, F)** represent the composition at the phylum level and genus level in PSO model mouse and CON, respectively. **(G)** GraPhlan plot showing dominant taxa in PSO model mouse. **(H)** Results of metagenomeSeq difference analysis in PSO model mouse. **(I)** Heat map of correlation between different genera of bacteria and laboratory indicators. HCs, Healthy Controls; PSO, Psoriasis patients and psoriasis model mouse; CON, Control Mouse; Hb, Hemoglobin; ALT, Alanine aminotransfease; TBIL, Total bilirubin; DBIL, Direct bilirubin; IBIL, Indirect Bilirubin; ALB, Albumin; GGT, Glutamyltranspeptidase; TBA, Total bile acid; TG, Triglyceride; ApoA, Apolipoprotein A; BUN, Urea nitrogen; Cr, Creatinine; CO_2_CP, Carbon dioxide-combining power *: p < 0.05, **: p < 0.01.

Gut microbiome in all states (HCs, PSO) consisted of four dominant phyla: *Firmicutes*, *Proteobacteria*, *Actinobacteriota*, and *Bacteroidota* (**Figures 3A, B**). The gut microbiome in PSO patients is characterized by an increase in *Firmicutes* and *Actinobacteriota*, while *Bacteroidota* decreases, in agreement with previous descriptions (12). The dominant genera in the disease state include *Faecalibacterium*, *Megamonas*, *Prevotella*, and *Escherichia-Shigella* (**Figure 3C**). We also used LEfSe identified *Escherichia-Shigella* as an additional discriminatory feature of a healthy gut (**Figure 3D**).

Similar results were observed in animal experiments using PSO rats, with an increase in Firmicutes and a decrease in *Bacteroidota* (**Figure 3E**). The specific genera that increased in the PSO rats were *Lachnospiraceae_NK4A136_group*, *Bacteroides*, *Clostridia_vadinBB60*, and *Roseburia*, while *Alloprevotella*, *Muribaculaceae*, and *Alistipes*, *Prevotellaceae_UCG-001*, *Rikenellaceae_RC9_gut*, and *Clostridia_UCG-014* decreased significantly (**Figure 3F**). It is important to note that the microbiota identified in PSO patients and PSO model mice were not identical (**Figure 3G**).

Further analysis using metanomeSeq revealed that the relative abundance of *Escherichia-Shigella*, *Enterococcus*, *Clostridium_sensu_stricto_1 strain*, *Prevotellaceae_NK3B31_group*, and *[Eubacterium]_xylanophilum_group* increased significantly in PSO model mice, while *Muriaculum*, *Ruminococcus*, *Oscillospiraceae_NK4A214_group*, *Parabacteroides* and *Odoribacter* decreased significantly (**Figure 3H**). These findings suggest that *Escherichia-Shigella* may play a critical role in the dysregulation of the intestinal microecology in PSO patients.

We next performed a correlation analysis to assess the mechanism of gut microbiota in PSO, which showed that *Escherichia-Shigella* was positively correlated with serum levels of Total Bile Acids (TBA) and negatively with levels of Hemoglobin (Hb), Direct Bilirubin (DBIL), and Indirect Bilirubin (IBIL) in patients with PSO. *Odoribacter* was positively correlated with the blood sedimentation rate (ESR). Several other differential genera, including *Lachnospiraceae_ND3007_group*, *Lachnospiraceae_UCG-004*, *[Eubacterium]_eligens_group*, *Parabacteroides*, and *Ruminococcus* were found to be positively correlated with Total Bilirubin (TBIL), DBIL and IBIL (**Figure 3I**). BAs acts as a signaling molecule that differentially affects the gut microbiota and host immunity as well as metabolism. BAs can directly inhibit IL-17A production and block CCL20-mediated transport to ameliorate psoriasiform dermatitis with minimal toxicity (32). Our study found a significant reduction in serum TBA levels in PSO patients (**Supplementary Figure 1**), therefore, we hypothesized that *Escherichia-Shigella* may exert pathogenic effects by affecting the BAs metabolic pathway.

### 3.4 Predictive analysis of faecal microbial function and biomarker screening

**Figure 4:**
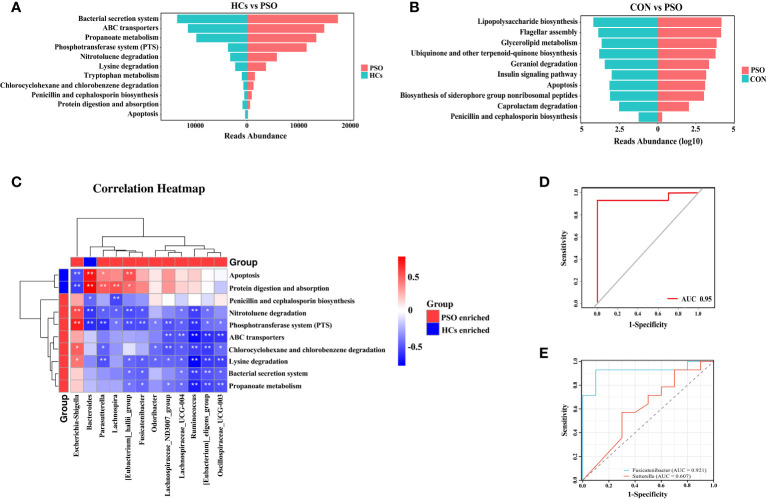
KEGG enrichment analysis and prediction of gut microbiome characteristics with machine learning classification algorithms. **(A)** KEGG enrichment analysis in PSO. **(B)** KEGG enrichment analysis in PSO model mice. **(C)** Correlation heat map revealing the relationship between differential genera and metabolic pathways in the gut of PSO patients. **(D, E)** Receiver operating characteristic curves using logistic regression for 2 genera. The diagonal line indicates the region under the curve value of 0.5, which is a random decision. HCs, Healthy Controls; PSO, Psoriasis patients and psoriasis model mouse; CON, Control Mouse *: p < 0.05, **: p < 0.01.

KEGG pathway enrichment analysis revealed enhanced pathways in PSO patients, including Lysine degradation, Phosphotransferase system (PTS), Nitrotoluene degradation, Penicillin and cephalosporin biosynthesis, Chlorocyclohexane and chlorobenzene degradation, Bacterial secretion system, Tryptophan metabolism, Propanoate metabolism, and ABC transport proteins. Some pathways, such as Protein digestion and absorption, and Apoptosis pathways, were attenuated (**Figure 4A**). Additionally, an attenuation of the Apoptosis pathway was observed in a mouse model of PSO (**Figure 4B**). These findings suggest that dysregulation of the gut microbiome, which leads to an attenuated apoptotic pathway, may play a crucial role in the pathogenesis of PSO.

Subsequently, through correlation analysis, we determined that the gut microbiome with high abundance in HCs exhibited a positive correlation with enhanced metabolic pathways in HCs. Specifically, *Bacteroides* showed a positive correlation with Protein digestion and absorption and Apoptosis pathways. In contrast, *Escherichia-Shigella*, enriched in PSO, demonstrated a negative correlation with Protein digestion and absorption and Apoptosis pathways, while exhibiting a positive correlation with PTS, Nitrotoluene degradation, Chlorocyclohexane and chlorobenzene degradation, and Lysine degradation (**Figure 4C**). Previous studies have reported that BAs can exacerbate skin inflammation by activating G-protein bile acid-coupled receptor 5 (TGR5) and sphingosine-1-phosphate (S1P) receptor 2 (S1PR2) in the skin (33). Therefore, we hypothesized that *Escherichia-Shigella* may facilitate the onset and progression of PSO by inhibiting apoptosis through the BAs metabolic pathway.

The diagnostic efficacy of potential discriminatory biomarkers for PSO was evaluated by ROC curve analysis. By employing Lasso regression analysis, a simplified model consisting of 13 microbial genera successfully discriminated between PSO patients and HCs. To enhance the diagnostic efficacy of PSO, we constructed a classifier based on a logistic regression model. Notably, when the two most important genera were included, the AUC reached 0.95, demonstrating high sensitivity (92.86%) and specificity (100%) (**Figure 4D**). Ultimately, *Fusicatenibacter* and *Sutterella* were identified as robust diagnostic markers for distinguishing PSO from HCs, with their respective areas under the curve being 0.9214 and 0.6071 (**Figure 4E**).

### 3.5 Psoriatic skin microbiota enriched with *Staphylococcus*

**Figure 5:**
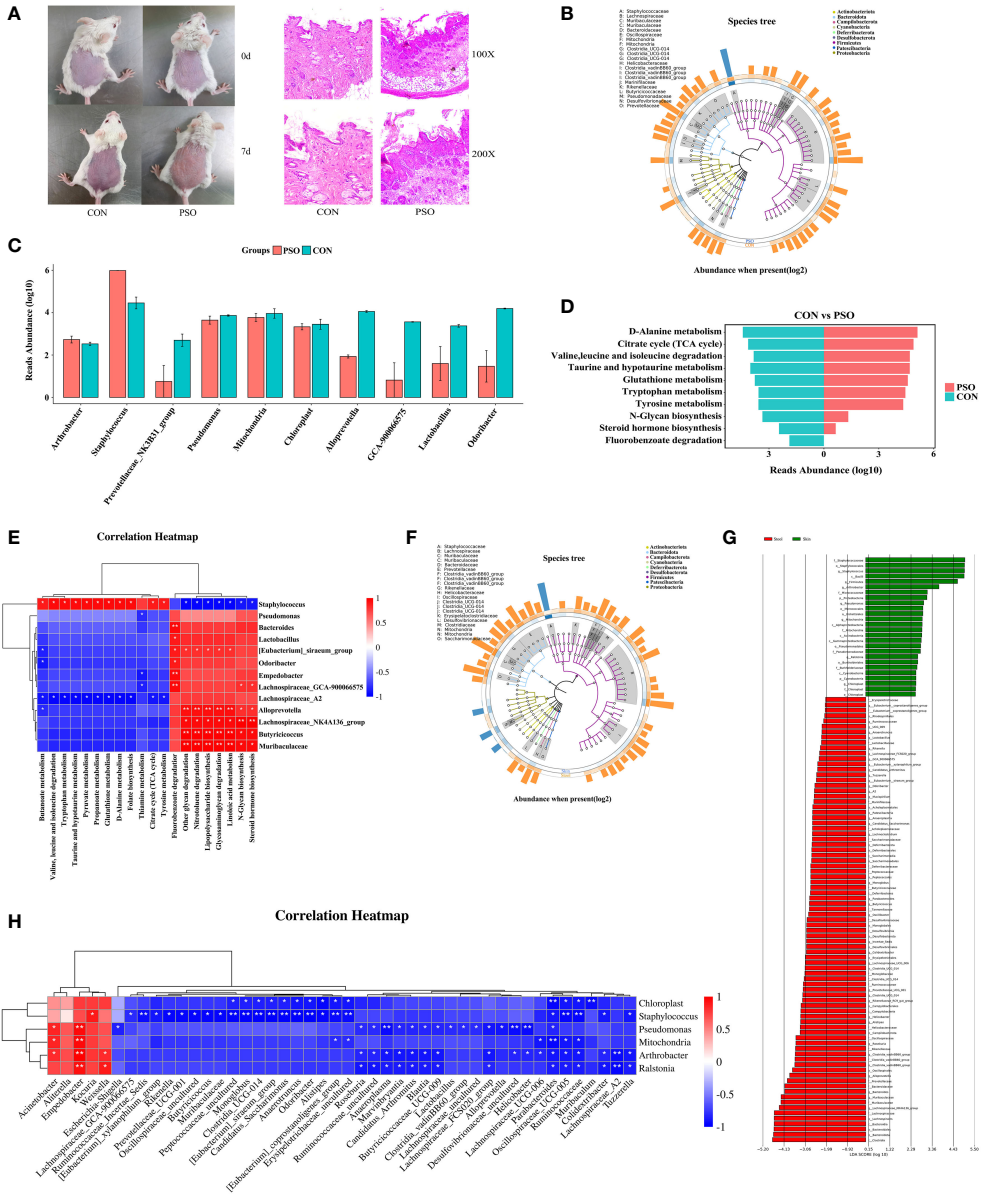
Skin microbiological characteristics of Psoriasis. **(A)** Diagram of the mouse model and representative micrographs of H&E staining of the skin. **(B)** GraPhlan plot showing the dominant flora in the skin of PSO model mouse. **(C)** Results of metagenomeSeq difference analysis showing only the top 10 genera with the most significant differences. **(D)** Enrichment analysis based on the results of metagenomeSeq difference analysis. The results of the metagenomeSeq difference analysis show only the top 10 genera with the most significant differences. **(E)** Correlation heatmap revealing the correlation between the differential genera and the enrichment pathway in the skin of PSO model mouse. **(F)** GraPhlan plot showing the dominant bacterial flora in the gut and in the skin of the PSO model. **(G)** LEfSe analysis revealing the difference between the gut and skin microbial communities in the PSO model mouse. **(H)** Correlation heatmap revealing the correlation between gut microbiota and skin microbials in the PSO model mouse. PSO, Psoriasis model mouse; CON, Control Mouse; Skin, Skin tissue of model mouse; Stool, Model mouse feces *: p < 0.05, **: p < 0.01.

PSO is an immune-mediated inflammatory skin disease, so it is particularly important to study the characterization of skin microecology in PSO. In this study, we investigated the composition of the skin microbiota in a PSO mouse model (**Figure 5A**). Our analysis revealed a significant increase in the phylum *Firmicutes* and a significant decrease in the phylum *Bacteroidota* in PSO model mice, in agreement with previous descriptions (34). At the genus level, *Staphylococcus* was found to dominate the skin microbiome in PSO model mice (**Figure 5B**). MetagenomeSeq difference analysis further identified *Staphylococcus* as a discriminatory feature between diseased and healthy skin (**Figure 5C**). These findings are consistent with previous literature reports, suggesting that *Staphylococcus* is the main causative genus responsible for the microecological dysbiosis of the skin in PSO patients (35).

Furthermore, KEGG enrichment analysis based on the metagenomeSeq results revealed the degradation of metabolic pathways such as Steroid hormone biosynthesis, Fluorobenzoate degradation, and N-Glycan biosynthesis in PSO model mice. On the other hand, Citrate Cycle (TCA cycle), Valine, leucine and isoleucine degradation, Tyrosine metabolism, Tryptophan metabolism, Taurine and hypotaurine metabolism, D-Alanine metabolism, and Glutathione metabolism were enhanced (**Figure 5D**). Correlation analysis showed that *Staphylococcus* is involved in multiple metabolic pathways, suggesting its contribution to skin lesions in PSO patients by affecting these pathways (**Figure 5E**).

The disruption of gut and skin microecology can have a significant impact on the overall homeostasis of the skin. Imbalances within the microbial community can either positively or negatively affect the integration of the gut and skin (20, 36). To evaluate the effect of an imbalance in the gut microbiome on the skin microecology, we compared the gut and skin microbiomes of mice from the same psoriasis (PSO) model. Our analysis revealed that the gut microbiome was more diverse than the skin microbiome (**Supplementary Figure 1A**). Additionally, the skin microbiota exhibited a more severe imbalance between *Firmicutes* and *Bacteroidota* (**Supplementary Figure 2B**). At the genus level, we observed that *Staphylococcus*, *Chloroplast*, *Mitochondria*, and *Pseudomonas* were dominant in the lesional skin of PSO model rats (**Supplementary Figure 2C**). However, the gut microbiome was dominated by *Lachnoclostridium*, *Rikenellaceae_RC9_gut_group*, *Clostridia_vadinBB60_group*, *Muribaculaceae*, *Butyricoccaceae_UCG-009*, *Parabacteroides*, *Alloprevotella*, *Lachnospiraceae_FCS020_group*, *Clostridia_UCG-014*, *Candidatus_Saccharimonas*, *Bacteroides*, *Candidatus_Arthromitus*, *Anaeroplasma*, *[Eubacterium]_siraeum_ group*, and *Prevotellaceae_NK3B31_group* (**Figure 5F**). Further analysis using LEfSe identified *Staphylococcus*, *Arthromitus*, *Pseudomonas*, *Mitochondria*, *Ralstonia*, and *Chloroplast* as discriminatory features of diseased skin, which includes many opportunistic pathogens (**Figure 5G**). This dysregulation of the skin microecology in PSO patients may compromise the skin barrier and lead to increased susceptibility to opportunistic pathogen infections. LEfSe also identified several discriminative features of intestinal microecological dysregulation in PSO, including *Lachnospiraceae_NK4A136_group*, *Muribaculaceae*, *Bacteroides*, *Alloprevotella*, *Clostridia_vadinBB60_group*, *Roseburia* (**Figure 5G**), which could potentially serve as markers for distinguishing between different tissues affected in PSO.

The understanding of the interplay between gut and skin microbiota can offer valuable insights for further investigations into PSO-related microbial communities, as microbial interactions may serve as the primary driving force behind the imbalance in these communities. By correlating the abundance of microorganisms, we explored the relationships between different microorganisms. Our analysis revealed a positive correlation between *Staphylococcus*, and *Kocuria*, *Empedobacter*, and *Weissella* at the genus level (**Figure 5H**). Moreover, we observed that the enrichment of *Pseudomonas* on the skin was positively associated with *Acinetobacter* and *Empedobacter* in the gut, while negatively correlated with various genera in the gut, including *Desulfovibrionaceae_uncultured, Helicobacter, Anaeroplasma, Alloprevotella, Lachnospiraceae_FCS020_group, Escherichia-Shigella* and *Blautia* (**Figure 5H**). Overall, our data suggest that disturbances in the gut microbial community may promote the colocalization of the skin by opportunistic pathogenic microorganisms such as *Staphylococcus* and *Pseudomonas*, exacerbating the disruption of skin microbiome in PSO and further contributing to the skin lesions in PSO.

### 3.6 IL-17A inhibitor modulates the gut microbiome in Psoriasis patients

**Figure 6:**
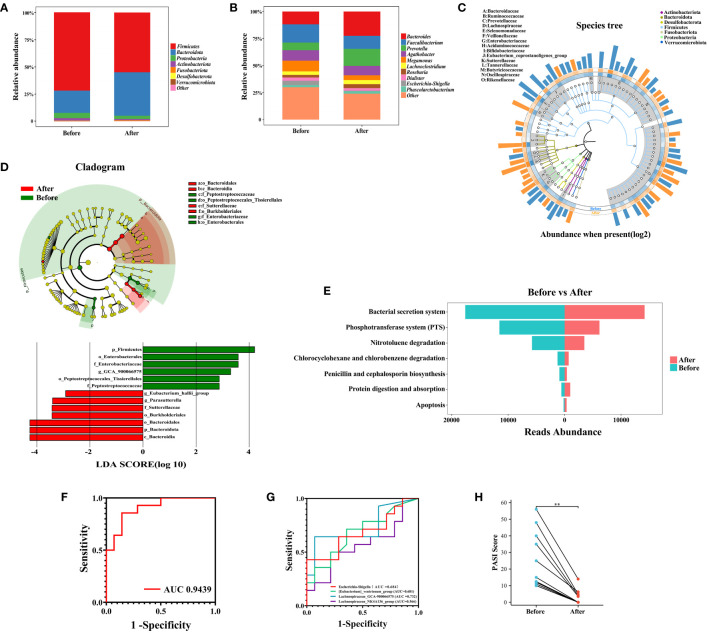
Changes in the gut microbiome before and after IL-17A inhibitor treatment. **(A)** and **(B)** Composition of gut microorganisms at the phylum and genus level before and after IL-17A inhibitor treatment. **(C)** GraPhlan plot showing the dominant flora in the gut before and after IL-17A inhibitor treatment. **(D)** Results of LEfSe analysis. **(E)** Results of KEGG enrichment analysis. **(F)** and **(G)** Receiver operating characteristic curves using logistic regression for 4 genera. **(H)** Pairwise plot showing a significant decrease in PASI scores in PSO patients after treatment with IL-17A inhibitor. Before, Before IL-17A inhibitor treatment; After, After IL-17A inhibitor treatment **: p < 0.01.

Th17 cells release IL-17A, which effectively triggers the activation of keratinocytes either alone or in combination with TNF. These keratinocytes subsequently overproduce inflammatory cytokines and chemokines, attracting and activating immune cells. This process establishes a positive feedback loop of sustained inflammation, ultimately leading to skin damage in PSO. Numerous studies have demonstrated the efficacy, rapid action, safety, and long-lasting benefits of Skuticilumab, a monoclonal antibody that inhibits IL-17A. It provides comprehensive advantages for treating moderate-to-severe Psoriasis (26, 37). IL-17 production by γδT cells is inhibited in a histone deacetylase-dependent manner, mediated by Short-chain fatty acids (SCFAs) derived from gut microbiota, particularly propionic acid. These SCFAs exert anti-inflammatory effects (38). The use of IL-17 inhibitors in PSO patients is associated with changes in the composition of gut microbiome (39). Although our findings did not reveal significant alterations in gut microbial diversity before and after Secukinumab treatment (**Supplementary Figure 3**), further intergroup comparisons demonstrated a decrease in the relative abundance of *Firmicutes, Proteobacteria, and Actinobacteriota*, while that of *Bacteroidota* significantly increased (**Figure 6A**) following Secukinumab treatment. At the genus level, Secukinumab treatment led to an increase in the relative abundance of *Bacteroides, Prevotella, Roseburia, Lachnoclostridium*, and *Phascolarctobacterium*, while *Faecalibacterium, Agathobacter, Megamonas, Dialister*, and *Escherichia-Shigella* decreased (**Figure 6B**). These findings suggest that Secukinumab treatment improves gut microbiota dysbiosis in PSO patients (**Figure 6C**).

To investigate the impact of IL-17 inhibitors on the gut microbiome, the LEfSe assay was utilized to identify the key changes in the bacterial composition of PSO patients treated with Secukinumab. After 16 weeks of treatment, the relative abundance of certain bacterial genera in fecal samples showed significant alterations. Specifically, there was a notable increase in *Parasutterella* and *Eubacterium_hallii_group*, while the abundance of *Lachnospiraceae_GCA_900066575* decreased (**Figure 6D**). Additionally, through PICRUSt2 functional predictive analysis, it was determined that anti-IL17 intervention in the gut microbiota of PSO patients can lead to changes in various metabolic pathways (40). Notably, Secukinumab treatment had a significant impact on pathways related to bacterial pathogenesis and virulence, such as the Bacterial secretion system, Phosphotransferase system (PTS), and Penicillin and cephalosporin biosynthesis, which were attenuated the most. Conversely, pathways associated with Apoptosis and Protein digestion and absorption showed the strongest increase after Secukinumab treatment (**Figure 6E**). These findings suggest that IL-17 inhibitor treatment alters the gut microbiota and its metabolites in PSO patients, bringing them closer to the composition seen in healthy individuals.

To identify potential biomarkers for assessing the efficacy of IL-17 inhibitors in patients with psoriasis, we utilized Lasso regression with logistic regression to analyze differential gut microbiota. Our analysis revealed that the inclusion of *Lachnospiraceae_GCA-900066575, Escherichia-Shigella, [Eubacterium]_ventriosum_group*, and *Lachnospiraceae_NK4A136_group* resulted in an area under the curve (AUC) of 0.9439 (**Figure 6F**), with corresponding AUC values of 0.732, 0.684, 0.681, and 0.566 (**Figure 6G**), respectively. Notably, all patients in our cohort experienced substantial remission of skin lesions upon Secukinumab treatment (**Figure 6H**). Therefore, we propose that these four gut microbiomes exhibit potential as biomarkers for evaluating the effectiveness of IL-17 inhibitors in PSO patients.

## 4 Discussion

In this study, we followed a rigorous sampling protocol and combined longitudinal cohort studies and animal experiments to measure the microbiome in the gut and skin by 16S rRNA. Animal experiments were conducted to validate the initial screening results and gain a deeper understanding of the microbiome associated with PSO. Our findings indicate that the gut microbiome in PSO patients exhibits higher heterogeneity. It is well known that impaired intestinal barrier function, increased inflammatory mediators, and release of metabolites can mediate the relationship between inflammatory skin disease and the gut microbiome (9). Most studies have shown no significant differences in α-diversity and β-diversity between HCs and PSO patients (9), which is consistent with our findings. However, some studies have reported severe ecological dysbiosis, lower diversity of the gut microbiota, and changes in the relative abundance of certain bacterial taxa in PSO patients (41). In line with these studies, we observed a significant increase in the phylum of *Firmicutes* and *Actinobacteriota*, as well as a significant decrease in the *Bacteroidota* phylum (41, 42). LEfSe analysis revealed that *Escherichia-Shigella* was significantly enriched in PSO patients. Correlation analysis showed a positive correlation between *Escherichia-Shigella* and serum levels of TBAs, as well as a negative correlation with levels of bilirubin. Previous studies have reported fat damage in PSO skin, characterized by lower concentrations of primary and secondary bile acids (43, 44), which aligns with our findings (**Supplementary Figure 3**). It has also been reported that changes in *Escherichia-Shigella* after Roux-en-Y gastric bypass (RYGB) surgery correlate with changes in BAs (45). Therefore, we hypothesize that *Escherichia-Shigella* in the intestine of PSO patients may promote the conversion of BAs into various forms through fecal excretion, leading to reduced BAs levels.

We also found a negative correlation between *Escherichia-Shigella* enrichment and the attenuated apoptotic pathways in PSO during functional enrichment analysis, suggesting that *Escherichia-Shigella* enrichment is involved in attenuated apoptotic pathways in PSO patients. Apoptosis is a specific form of cell death that usually prevents inflammation and tissue damage (46). This can result in the inhibition of Extracellular signal regulated kinase (ERK1/2) and phosphoinositide-3 kinase (PI3K)/serine/threonine protein kinase (Akt) signaling pathways by Sphingosine-1-phosphate receptor 2 (S1PR2), leading to the overproliferation of psoriatic keratinocytes and the development of PSO skin lesions (47, 48). Psoriatic keratinocytes in lesions exhibit increased resistance to apoptosis, which disrupts epidermal homeostasis and contributes to the pathogenesis of PSO (49, 50). Microarray transcriptome analysis of psoriatic lesions has revealed the upregulation of anti-apoptotic gene expression and the downregulation of pro-apoptotic gene expression (51). Sphingosine-1-phosphate (S1P), a bioactive sphingolipid, is involved in various cellular processes, including cell proliferation, differentiation, inflammation, and malignant transformation. S1P inhibits macrophage migration to inflammatory sites through the activation of S1PR2 and promotes wound healing (52–54). BAs, as upstream activators of S1PR2, can directly modulate S1PR2 activity and mediate the S1PR2 pathway through the Farnesol X Receptor (FXR) and TGR5 signaling pathways. Conjugated bile acids (CBAs) can activate the ERK1/2 and Akt signaling pathways via S1PR2, exerting an inhibitory effect on apoptosis (55–57). Therefore, the dysregulation of the gut microbiome, specifically the elevation of *Escherichia-Shigella*, may disrupt BAs metabolism, leading to the inhibition of ERK1/2 and Akt signaling pathways by S1PR2. This dysregulation ultimately promotes the overproliferation of keratinocytes and the development of PSO skin lesions.

Recognition of microbial-associated molecular patterns (MAMP) through specific pattern recognition receptors (PRRs), interactions between symbiotic organisms and hosts (58). Keratinocytes produce the antimicrobial peptide pepsin (LL-37) through contact with symbiotic microorganisms, which bind to nucleic acids in epithelial cells and stimulate the production of type I interferons, while their own RNA interacts with LL-37 to induce nitric oxide synthase (iNOS) to produce TNF-α (59). These cytokines influence the differentiation of primitive T cells into Th17 cells, which produce the interleukins IL-17 and IL-22, leading to the development of psoriatic lesions (60). Furthermore, Malassezia in the skin can metabolize to produce oleic acid (61) and the amount of oleic acid in the lesional skin of patients with psoriasis correlates with a reduction in IL-17 driver signatures following T cell activation (62), suggesting that skin micro ecological dysregulation compromises the skin barrier and alters skin composition, which in turn affects local immune function and promotes the development of psoriatic lesions. In line with previous studies, we noted a decrease in relative abundance of *Bacteroides* and *Proteobacteria* associated with PSO skin, while an increase in relative abundance of *Firmicutes* (63). Multiple studies have reported increased colonization of *Staphylococcus* in psoriatic skin (64). We found a significant increase in *Staphylococcus* colonization in both psoriatic lesion and non-lesion sites compared to healthy skin. Additionally, neonatal mice colonized with *Staphylococcus* exhibit strong Th17 polarization, leading to increased production of IL-17A and exacerbation of skin lesions (35). These findings suggest that the increased presence of *Staphylococcus* is not simply a result of structural changes in the skin caused by psoriasis, but rather an important trigger for disease progression. Furthermore, our analysis revealed that *Staphylococcus* colonization is associated with several metabolic pathways that are enhanced in psoriasis, including the TCA cycle, Tryptophan metabolism, Butanoate metabolism, and other Amino Acid metabolic pathways. *Staphylococcus* may play a regulatory role in energy homeostasis through its involvement in the TCA cycle. Citrate, the first intermediate of the TCA cycle, binds and activates catabolic control protein E (CcpE) (65), a major regulator of *Staphylococcus* virulence factor expression, central metabolism, iron acquisition, and bacterial virulence. Modulation of TCA cycle activity may provide *Staphylococcus* with a survival advantage during infection (66, 67). Inactivation of aconitase (CitB) leads to downregulation of the TCA cycle, thereby preventing maximal expression of virulence factors and altering the interaction between *Staphylococcus* and its host (68–70). Therefore, we hypothesize that *Staphylococcus* may exert its virulence effects by modulating the activity of the TCA cycle, ultimately contributing to the development of psoriatic skin lesions.

Gut microecological dysregulation can negatively impact skin integrity and function (71). Mucosal immune elements and signaling pathways play a crucial role in coordinating epidermal differentiation and intestinal barrier function (72, 73). Additionally, the transmission of intestinal microbes and their metabolites to the skin has been shown to impact skin physiology and the immune system (74). For instance, metabolites like cresol and phenol produced by *Clostridium difficile*, which are biomarkers of intestinal microbial dysbiosis, enter the bloodstream and accumulate in the skin, leading to compromised skin barrier integrity and impaired epidermal differentiation, thereby affecting keratinization (75). In our study, we observed significant differences in the gut microbiome and skin microbiome of PSO patients. *Staphylococcus* and *Mitochondria* were the dominant genera in the skin microbiome, whereas the gut microbiome was characterized by dominant genera such as *Lachnospiraceae, Muribaculaceae, Bacteroidaceae, Prevotellaceae, Clostridia vadinBB60 group, Rikenellaceae*, and *Helicobacteraceae.* Correlation analysis revealed that *Staphylococcus* was correlated with a wide range of genera in the gut, including SCFAs producers like *Butyricicoccus, Monoglobus, Clostridia_UCG_014, Prevotellaceae_UCG_001*, *Parabacteroides*, etc. SCFAs can regulate energy metabolism and immunity, suppress inflammatory responses, stabilize the intestinal ecosystem and maintain intestinal integrity. Sodium butyrate significantly down-regulates mRNA expression of biofilm-forming associated gene aggregation factor B (ClfB) and serine aspartate repeat (SdrC) of protein C, thereby inhibiting staphylococcal invasion (76). Butyrate and propionate ameliorate *Staphylococcus*-induced inflammatory responses by inhibiting NF-κB, IFN-β/STAT1, and HDAC, thereby reducing NO production (77). Based on these findings, we hypothesize that the reduction of SCFAs-producers in the gut of PSO patients may promote *Staphylococcus* colonization on the skin and accelerate the onset and progression of PSO.

In young individuals, PSO can often be mistaken for Atopic Dermatitis (AD) (78). AD lesional skin typically exhibits low bacterial diversity (79), accompanied by a significant increase in *Staphylococcus aureus* (80), which aligns with our observations in PSO lesional skin. Recent research has identified dysbiosis of the gut microbiota as a key factor in the pathogenesis of AD. Multiple studies have demonstrated that dysbiosis of the gut microbiota, characterized by a reduction in SCFAs-producing bacteria, plays a pivotal role in the development of AD (81). For instance, *Clostridium, Prevotella, Clostridia, Bifidobacteraceae, Bifidobacterium, Bacteroidetes, Christensenellaceae_R7_group*, and other bacterial species have been found to be decreased (82–85). Additionally, the “gut-skin” axis has emerged as a novel therapeutic target for the prevention and management of AD.

In mouse models, the skin microbiome, specifically *Staphylococcus* (35), can enhance Th17 polarization to promote PSO-like skin inflammation (86), while immunotherapy can alter the composition and classification of the gut microbiome and lead to changes in gut metabolism and immune response regulation (87, 88). IL-17 cytokine production effectively activates keratin-forming cells and promotes inflammation in the feed-forward circuit. The IL-17 pathway plays a key pathogenic role in autoimmune and inflammatory diseases and has been the focus of targeted therapies. Secukinumab is a selective IL-17A inhibitor that has been approved for PSO, Psoriatic Arthritis (PsA) in adults and children (26, 37). Our data show that the gut microbiome of PSO patients after Secukinumab treatment tends to change toward the distribution of normal human flora. *Parasutterella* and *Eubacterium_hallii_group* were the dominant bacteria found in the intestines of patients with PSO after treatment with the Secukinumab. *Parasutterella* is a type of Betaproteobacteria and is normally present in the healthy human gastrointestinal tract (89). Previous studies have shown that a high-fat diet (HFD) and *Clostridium difficile* infection (CDI) can decrease the abundance of *Parasutterella* in the gut (90, 91). However, an increase in *Parasutterella* has been found to have a protective effect against Intrahepatic Cholangiocarcinoma (ICC) (92). *Parasutterella* produces succinic acid and hypoxanthine (93), and is negatively correlated with levels of faecal Deoxycholic Acid (DCA) and Lithocholic Acid (LCA) (94). Hypoxanthine plays a key role in maintaining intestinal barrier function (95). On the other hand, *Eubacterium_hallii_group* can ameliorate intestinal inflammation and regulate BAs metabolism in the host through the production of SCFAs (96). Based on these findings, it is hypothesized that IL-17A inhibitors may improve intestinal inflammation in PSO patients by increasing the abundance of *Eubacterium_hallii_group*, which promotes SCFA production while increased *Parasutterella* promotes hypoxanthine production and regulates BAs metabolism in patients, which in turn improves intestinal barrier function and achieves the relief of clinical symptoms. In addition, the increase in Parasutterella after IL-17A inhibitor therapy may be related to the development of inflammatory bowel disease in some PSO patients (97), as higher levels of *Parasutterella* are associated with chronic inflammation in Irritable Bowel Syndrome (IBS) (98). Further research is needed to understand the specific mechanism of *Parasutterella* in IL-17A inhibitor therapy.

KEGG enrichment analyses revealed a significant enhancement of the previously attenuated Protein digestion and absorption and apoptosis pathways. Impaired lipolysis characterized by low concentrations of BAs is present in psoriatic skin. About one-third of patients with PSO eventually convert to PsA, and results from untargeted metabolomics studies have shown that patients with PsA have reduced serum BAs and Butyrate levels and increased levels of inflammatory lipid mediators (43, 99). Blocking the transport mediated by CCL20, a chemokine involved in PSO, can be improved by gut microbiota-derived BAs (32). Gut microbiota-derived SCFAs, which inhibit IL-17 production by intestinal γδ T cells (38), and *in vitro* supplementation with *Bifidobacterium short CCFM683* improved PSO-like dermatitis by restoring the intestinal microbiome of PSO model rats, promoting BAs production, modulating the FXR/NF-κB pathway, reduces the generation of inflammatory cytokines, modulating keratinocytes and maintaining epidermal barrier function to attenuate PSO (100). IL-17 accumulation in lesions is a key factor in PSO pathogenesis, and inhibiting IL-17 production can attenuate abnormal keratinocyte proliferation. *Staphylococcus* colonization in PSO patients’ epidermis promotes IL-17A expression (35), which delays wound healing and can be abrogated by IL-17-blocking monoclonal antibodies (101–103). Therefore, using IL-17A inhibitors may inhibit *Staphylococcus* proliferation, reduce IL-17 expression, promote skin lesion healing, and restore the gut microbiome through the “gut-skin” axis. This can increasing *Parasutterella* and *Eubacterium_hallii_group*, promoting SCFAs and BAs production, reducing pro-inflammatory cytokine expression, regulating keratinocyte apoptosis, restoring the microbiota and epidermal barrier function, and improving PSO symptoms. In addition, *Fusicatenibacter* and *Sutterella* can serve as potential biomarkers for PSO diagnosis, while *Lachnospiraceae_GCA-900066575, Escherichia-Shigella, [Eubacterium]_ventriosum_group*, and *Lachnospiraceae_NK4A136_group* have potential value in discriminating PSO disease remission. This provides a rationale for targeting gut microbiome alterations to treat PSO.

## 5 Conclusion

In this investigation, we discovered that PSO is characterized by alterations in the intrinsic microbial communities of the gut and skin, as well as disruptions in their metabolic pathways. This was determined through longitudinal studies and validation of mouse models, suggesting that the interactions between gut and skin microbes may play a crucial role in the development of PSO. Interestingly, the administration of IL-17A inhibitors restored the gut microbiota of PSO patients, potentially leading to an improvement in their clinical symptoms. This finding highlights the importance of maintaining a healthy gut microbiota for individuals with PSO and supports the connection between the gut-skin axis and PSO. It is noteworthy that this study is the first to observe an increase in *Parasutterella* in the gut of PSO patients after IL-17A inhibitor treatment. The bidirectional effect of *Parasutterella* on gut inflammation may serve as a possible mechanism by which IL-17A inhibitors contribute to inflammatory bowel disease. However, further investigation is required to better understand the role of *Parasutterella* in the gut of PSO patients. It should be noted that this study has limitations, as it involved a small sample size. Therefore, additional research is needed to confirm the causal relationship between gut microbiota and PSO, and these findings should be validated in larger studies.

## Data availability statement

The data presented in the study are deposited in the Sequence Read Archive (SRA) repository, accession number PRJNA1078442.

## Ethics statement

The studies involving humans were approved by the Ethics Committee of Heji Hospital of Changzhi Medical College (number: 2023-13). The studies were conducted in accordance with the local legislation and institutional requirements. The participants provided their written informed consent to participate in this study. The animal study was approved by the Ethics Committee of Changzhi Medical College (number: DW2023051). The study was conducted in accordance with the local legislation and institutional requirements.

## Author contributions

HZ: Funding acquisition, Resources, Writing – review & editing, Conceptualization. LS: Formal Analysis, Writing – original draft, Data curation, Writing – review & editing. YZ: Data curation, Writing – original draft. ZL: Investigation, Writing – original draft. NW: Investigation, Writing – original draft. QZ: Resources, Writing – original draft. CG: Methodology, Writing – review & editing. JL: Funding acquisition, Project administration, Supervision, Writing – review & editing, Conceptualization.
