# Supplementary material for: IL-17A inhibitors alleviate Psoriasis with concomitant restoration of intestinal/skin microbiota homeostasis and altered microbiota function
Source: Front Immunol. 2024 Feb 28;15:1344963. doi: 10.3389/fimmu.2024.1344963 (PMC10933079; doi:10.3389/fimmu.2024.1344963)
Supplement: Supplementary file 1 [file DataSheet_1.docx]

Supplementary Material

## 1 Supplementary Figures


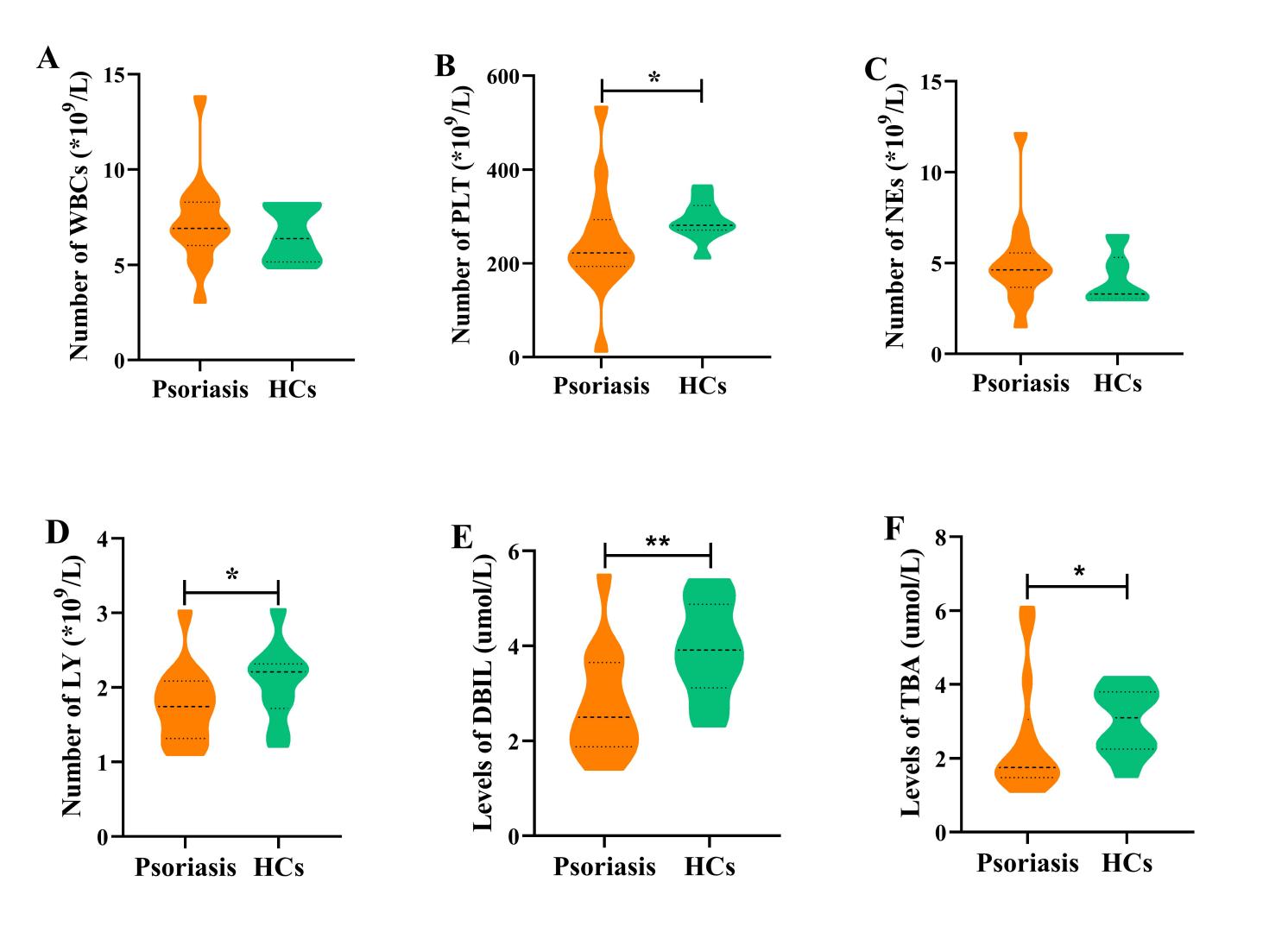


**Supplementary Figure 1. General laboratory results in patients with psoriasis.** (A) No significantly different WBC levels were seen in patients with psoriasis. (B) PLT levels were significantly lower in patients with psoriasis. (C) No significant difference in NE levels was seen in patients with psoriasis. (D) Lymphocyte counts, (E) DBIL levels, and (F) TBA levels were reduced in patients with psoriasis compared to normal subjects. HCs: Healthy Controls. WBC: White Blood Cell. PLT: Blood Platelet. NE: Neutrophil. LY: Lymphocyte. DBIL: Direct Bilirubin. TBA: [Total Bile Acid](file:///D:/%E7%99%BE%E5%BA%A6%E7%BF%BB%E8%AF%91/baidu-translate-client/resources/app.asar/app.html#/#). *: *p* < 0.1, **: *p* < 0.05. WBC: White blood cell, PLT: Platelet, NEs: Neutrophile, LY: Lymphocyte, DBIL: Direct bilirubin, TBA: Total bile acid.

**
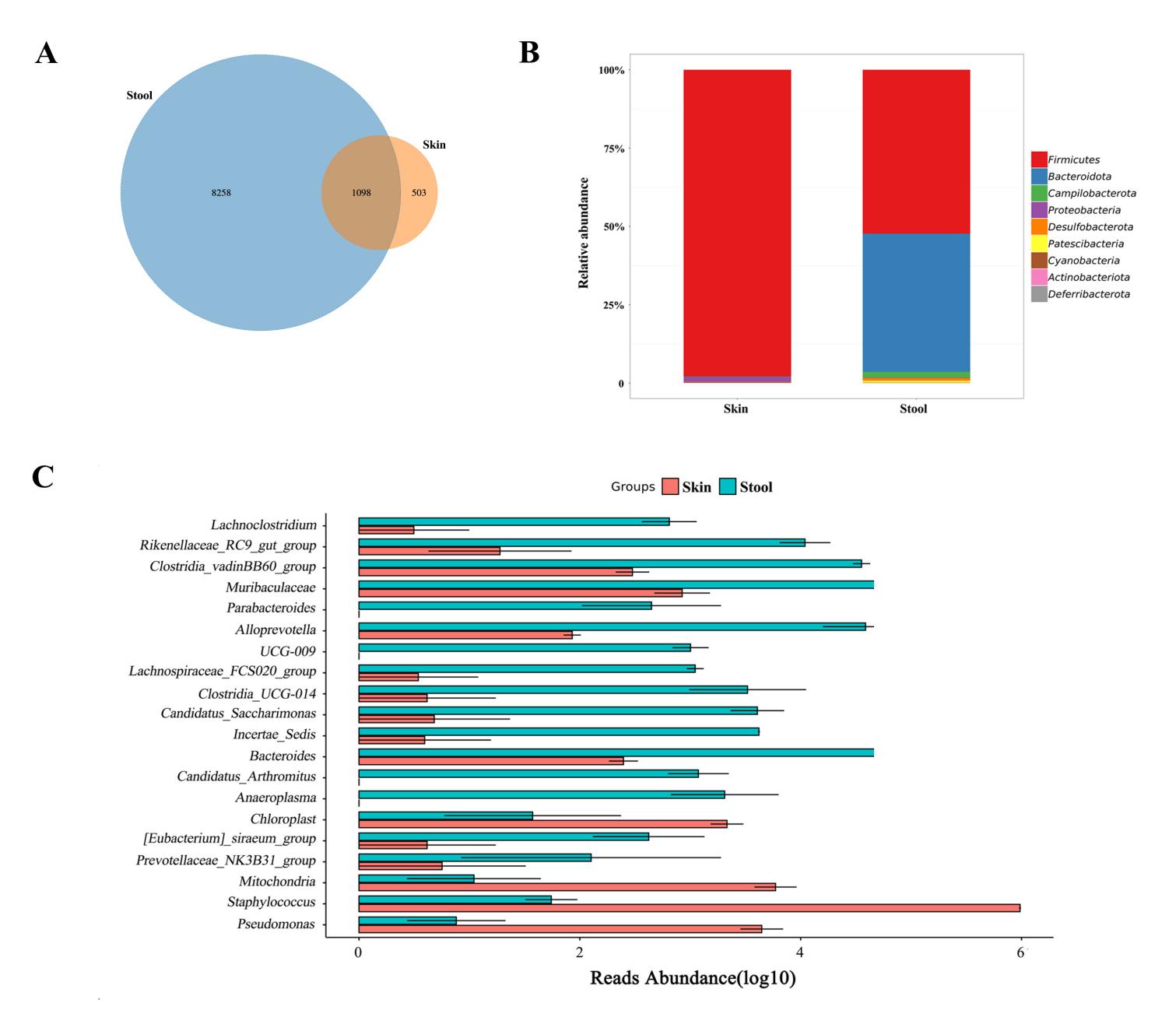
**

**Supplementary Figure 2.** Differences between the gut microbiome and skin microbiome in psoriasis. (A) Wayne diagram showing the number of OTUs in the gut microbiome and skin microbiome in psoriasis. (B) Gate and (C) genus level composition of the gut microbiome and skin microbiome in psoriasis.


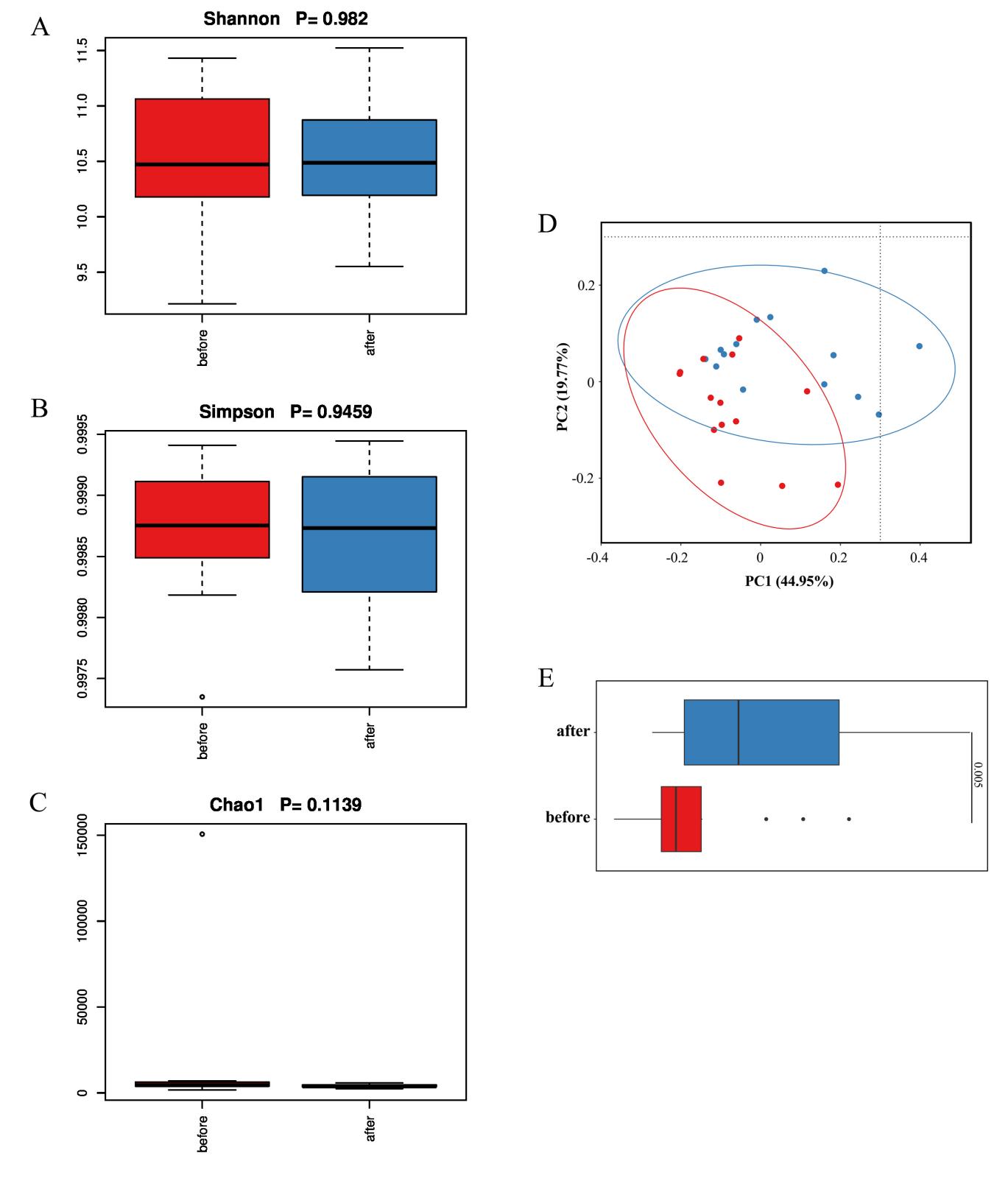


**Supplementary Figure 3.** **Bacterial community diversity in the psoriatic gut before and after IL-17A inhibitor treatment.** Alpha diversity measured according to (A) Shannon index, (B) Simpson’s diversity index, and (C) chao1 index of healthy skin samples. (D) Principal coordinate analysis (PCoA) of the microbial community structures based on weighted UniFrac distance matrix for the first two principal axes. Each point on the PCoA plot represents a fecal microbiome sample (red = before, blue = after). The first principal coordinate explains 44.95% of variation, and the second principal coordinate explains 19.77% of the variation. (E) The average weighted UniFrac distances among samples within each state are shown in the box plot.

**2 Supplementary Tables**

***Supplementary Table 1:* Clinical characteristics of the study participants**

|  | Psoriasis (n=14) | Healthy Controls (n=10) | P value |
| --- | --- | --- | --- |
| WBC (*10^9^/L) | 7.23 ± 2.46 | 6.57 ± 1.39 | 0.448 |
| Hemoglobin (g/L) | 148.86 ± 16.52 | 147.7 ± 16.57 | 0.867 |
| PLT (*10^9^/L) | 246.8 ± 119.49 | 291.4 ± 43.83 | 0.274 |
| Neutrophile (*10^9^/L) | 4.98 ± 2.48 | 4.15 ± 1.42 | 0.352 |
| Lymphocyte (*10^9^/L) | 1.76 ± 0.51 | 2.08 ± 0.53 | 0.157 |
| AST (U/L) | 34.67 ± 28.20 | 22.27 ± 9.47 | 0.197 |
| ALT (U/L) | 30.28 ± 16.39 | 21.09 ± 5.65 | 0.105 |
| AST/ALT | 0.91 ± 0.37 | 1.09 ± 0.31 | 0.234 |
| TBIL (g/L) | 13.45 ± 2.54 | 11.51 ± 4.74 | 0.260 |
| DBIL (μmol/L) | 2.75 ± 1.13 | 3.92 ± 1.01 | 0.016* |
| IBIL (μmol/L) | 9.39 ± 2.31 | 9.8 ± 1.0 | 0.607 |
| TP (g/L) | 76.4 ± 5.19 | 75.46 ± 3.18 | 0.618 |
| ALB (g/L) | 46.21 ± 2.34 | 46.77 ± 2.22 | 0.564 |
| GLO (g/L) | 31.59 ± 5.83 | 29.49 ± 4.54 | 0.352 |
| ALP (U/L) | 79.69 ± 18.78 | 80.75 ± 14.11 | 0.881 |
| GGT (U/L) | 40.28 ± 27.79 | 29.4 ± 4.21 | 0.235 |
| TBA (μmol/L) | 2.48 ± 1.63 | 3.01 ± 0.93 | 0.370 |
| TC (mmol/L) | 4.8 ± 1.86 | 4.96 ± 1.05 | 0.817 |
| TG (mmol/L) | 2.21 ± 1.01 | 1.98 ± 0.87 | 0.574 |
| HDL (mmol/L) | 1.42 ± 0.63 | 1.30 ± 0.24 | 0.567 |
| LDL (mmol/L ) | 3.18 ± 1.17 | 3.03 ± 1.0 | 0.743 |
| ApoA (g/L) | 1.29 ± 0.47 | 1.30 ± 0.22 | 0.942 |
| ApoB100 (g/L) | 1.14 ± 0.31 | 1.08 ± 0.22 | 0.636 |
| BUN (mmol/L) | 4.99 ± 1.50 | 4.17 ± 1.10 | 0.155 |
| Cr (mmol/L) | 67.29 ± 14.90 | 60.88 ± 15.12 | 0.313 |

Mean (x±s). Intergroup comparisons were made using the Independent-Samples T Test. WBC: White blood cell, PLT: Platelet, AST: Aspartate transaminase, ALT: Alanine aminotransfease, TBIL: Total bilirubin, DBIL: Direct bilirubin, IBIL: Indirect Bilirubin, TP: Total Protein, ALB: Albumin, GLO: Globulin, ALP: Alkaline Phosphatase, GGT: Glutamyltranspeptidase, TBA: Total bile acid, TC: Total cholesterol, TG: Triglyceride, HDL: High density lipoprotein, LDL: Low density lipoprotein, ApoA: Apolipoprotein A, ApoB100: Apolipoprotein B100, BUN: Urea nitrogen, Cr: Creatinine.

***Supplementary Table 2:* General information of** **Psoriasis patients with different clinical manifestations**

|  | Guttate Psoriasis (n=5) | Plaque Psoriasis (n=9) | HCs (n=10) | *P* value |
| --- | --- | --- | --- | --- |
| **Demographic parameters** |  |  |  |  |
| Age (years) | 39.80 ± 10.21 | 38.0 ± 11.15 | 49.10 ± 11.13 | 0.121 |
| Female / male, n | 2/ 3 | 2/ 7 | 7 / 3 | 0.109 |
| Disease duration (years) | 11.20 ± 3.96 | 16.11 ± 7.06 | - | 0.147 |
| Mean PASI | 11.80 ± 1.92 | 27.72 ± 17.66 | - | 0.112 |
| ESR (mm/h) | 7.0 ± 1.58 | 7.11 ± 1.9 | - | 1 |
| **Laboratory parameters** |  |  |  |  |
| WBC (*10^9^/L) | 6.80 ± 1.10 | 7.47 ± 3.01 | 6.57 ± 1.39 | 0.631 |
| PLT (*10^9^/L) | 307.2 ± 129.63 | 213.24 ± 106.07 | 291.4 ± 43.83 | 0.067 |
| Neutrophile (*10^9^/L) | 4.52 ± 1.18 | 5.23 ± 3.01 | 4.15 ± 1.42 | 0.665 |
| Lymphocyte (*10^9^/L) | 2.12 ± 0.53 | 1.56 ± 0.41 | 2.08 ± 0.53 | 0.04* |

Mean (x±s). Intergroup and Within-group comparisons of duration were made using the Independent-Samples T Test. Intergroup and Within-group comparisons of gentle and age were made using Pearson Chi-square test. ESR: Erythrocyte Sedimentation Rate, WBC: White blood cell, PLT: Platelet.

***Supplementary Table 3*: General information**

| Groups | n | Gentle | | Age （years） |
| --- | --- | --- | --- | --- |
|  |  | Male | Female | Mean±Std |
| Psoriasis | 14 | 10 | 4 | 38.64 ± 10.46 |
| Healthy controls | 10 | 3 | 7 | 49.10 ± 11.13 |
| χ ^2^ |  | 2.537 | |  |
| df |  | 1 | |  |
| *P* |  | 0.111 | | 0.091 |

Mean (x±s, years). Intergroup and Within-group comparisons of gentle and age were made using the Pearson Chi-square test and Independent-Samples T Test.
